# Dual-tracer PET/CT protocol with [^18^F]FDG and [^68^Ga]Ga-FAPI-46 outperforms single-tracer PET/CT with [^18^F]FDG in different cancer types, resulting in larger functional and gross tumor volume

**DOI:** 10.1007/s00066-023-02117-2

**Published:** 2023-08-16

**Authors:** Simone Wegen, Jasmin Weindler, Conrad-Amadeus Voltin, Lutz van Heek, Klaus Schomäcker, Thomas Fischer, Simone Marnitz, Carsten Kobe, Alexander Drzezga, Katrin S. Roth

**Affiliations:** 1 Department of Radiation Oncology, Cyberknife and Radiotherapy, Faculty of Medicine, University Hospital Cologne, Cologne, Germany; 2 Department of Nuclear Medicine, Faculty of Medicine, University Hospital Cologne, Kerpener Str. 62, 50937 Cologne, Germany

**Keywords:** Dual-tracer PET/CT, Gross tumor volume, Functional tissue volume, FDG, FAPI

## Abstract

**Purpose:**

Fibroblast activation protein (FAP) detected by positron-emission tomography (PET) using fibroblast activation protein inhibitor (FAPI) appears to be a promising target for cancer imaging, staging, and therapy, providing added value and strength as a complement to [^18^F]fluorodeoxyglucose (FDG) in cancer imaging. We recently introduced a combined single-session/dual-tracer protocol with [^18^F]FDG and [^68^Ga]Ga-FAPI for cancer imaging and staging. Malignant tissue visualization and target-to-background uptake ratios (TBRs) as well as functional tumor volume (FTV) and gross tumor volume (GTV) were assessed in the present study with single-tracer [^18^F]FDG PET/computed tomography (CT) and with dual-tracer [^18^F]FDG&[^68^Ga]Ga-FAPI-46 PET/CT.

**Methods:**

A total of 19 patients with head and neck and gastrointestinal cancers received initial [^18^F]FDG-PET/CT followed by dual-tracer PET/CT after additional injection of [^68^Ga]Ga-FAPI-46 during the same medical appointment (on average 13.9 ± 12.3 min after injection of [^18^F]FDG). Two readers visually compared detection rate of malignant tissue, TBR, FTV, and GTV for tumor and metastatic tissue in single- and dual-tracer PET/CT.

**Results:**

The diagnostic performance of dual-tracer compared to single-tracer PET/CT was equal in 13 patients and superior in 6 patients. The mean TBRs of tumors and metastases in dual-tracer PET/CTs were mostly higher compared to single-tracer PET/CT using maximal count rates (CRmax). GTV and FTV were significantly larger when measured on dual-tracer compared to single-tracer PET/CT.

**Conclusion:**

Dual-tracer PET/CT with [^18^F]FDG and [^68^Ga]Ga-FAPI-46 showed better visualization due to a generally higher TBR and larger FTV and GTV compared to [^18^F]FDG-PET/CT in several tumor entities, suggesting that [^68^Ga]Ga-FAPI-46 provides added value in pretherapeutic staging.

## Introduction

Malignant tumors mostly consist of two compartments: the tumor microenvironment and malignant cells. The transmembrane glycoprotein fibroblast activation protein (FAP) is highly expressed in the tumor microenvironment/stroma, cancer-associated fibroblasts (CAFs), and pericytes, accounting for the predominant portion of the tumor mass. However, it is not expressed directly on tumor cells, vascular epithelial cells, or inflammatory cells [1]. Several in vivo and histological studies found high expression of FAP in the majority of human epithelial malignancies [2–4]. Due to its increased expression in the stroma of tumors and of metastases, and given the potential impact of the tumor stroma on treatment outcome, FAP was identified as a new and promising target for tumor detection and therapy [5, 6]. Recent studies with quinolone-based positron-emission tomography (PET) tracers, acting as FAP inhibitors (FAPI), showed promising results with [^68^Ga]Ga-FAPI-PET for diagnosis and staging of various cancers [3, 7], with comparable or even better results in primary tumor detection than [^18^F]fluorodeoxyglucose(FDG)-PET/computed tomography (CT) [8]. Compared to [^18^F]FDG, [^68^Ga]Ga-FAPI shows a fast tumor uptake [8] with an equal or higher tumor to background ratio [9] and low uptake in most healthy organs including brain and liver, allowing the detection of malignant lesions in these organs [10, 11]. A recent study in patients with head and neck tumors (HNT) showed significantly larger gross tumor volumes (GTVs) measured with [^68^Ga]Ga-FAPI-46-PET/CT compared to [^18^F]FDG-PET/CT [12] and an accurate tumor delineation with [^68^Ga]Ga-FAPI-PET/CT, matching with tumor clipping [13]. In addition, a recently published study points to the potential of FAPI-PET/CT for response prediction in patients with esophageal cancer [14]. This is in line with a meta-analysis evaluating FAP expression in various tumors and showing significant correlation between high FAP expression and tumor progression [15].

As recently described, there is no gold standard for target volume delineation in esophageal cancer and parameters predicting a response to radiotherapy are missing [16]. For head and neck cancer, the target volume delineation of tumors with [^18^F]FDG-PET/CT has high concordance with the histopathological tumor extent, but the small superficial tumor parts and micrometastases are not reliably localized [16]. In esophageal cancer for example, the treatment decision between neoadjuvant versus definitive radiotherapy was shown to lead to significant differences in locoregional control and overall survival for patients with advanced disease [17]. Therefore, and since it was shown that chemoradiation can lead to changes in the immune cell composition and, consecutively, in the tumor microenvironment in cervical cancer [18], studies displaying and in the future treating different compartments of the tumor are necessary.

Due to tumor heterogeneity also given the different tumor compartments, exclusive imaging of the tumor microenvironment with [^68^Ga]Ga-FAPI-PET/CT may not be universally recommended or always sufficient in the diagnostic workup of malignant diseases. With regard to the different mechanisms of action and tracer retention, [^18^F]FDG and [^68^Ga]Ga-FAPI-PET/CT may represent complementary tools, capturing different aspects of tumor biology. Since [^18^F]FDG-PET/CT still represents a well-established standard for diagnostic imaging in many cancer types, and for guiding treatment planning, we recently established a single-session/dual-tracer PET/CT protocol for cancer staging prior to radiotherapy consisting of an [^18^F]FDG-PET/CT and a subsequent repeat scan following the injection of [^68^Ga]Ga-FAPI [19]. In a preliminary study testing this protocol, dual-tracer PET/CT demonstrated equal and, in some respects, superior performance in lesion detection compared to each single-tracer PET/CT.

The aim of the present study was to confirm the feasibility and explore the potential of dual-tracer PET/CT by investigating the performance of dual-tracer PET/CT compared to single-tracer PET/CT for malignant tissue detection in a larger cohort. To assess differences in calculated tumor volume measurements between single- and dual-tracer protocols for staging and treatment planning, we evaluated GTV and FTV measurements in patients with head and neck tumors (HNT) and esophageal cancer in both scans.

## Materials and methods

### Patient cohort, dual-tracer protocol, and PET/CT imaging

The retrospective analysis included 19 patients with head and neck and gastrointestinal cancer with a single-session/dual-tracer PET/CT protocol for cancer staging prior to radiotherapy without any known diseases of brain or liver, who received their PET/CTs in the period from September 2021 to March 2022. Dual-tracer PET/CT imaging was carried out according to a previously published protocol [19]. PET/CT scans were acquired in a supine position in craniocaudal direction. In 17 patients, the entire brain was included in the PET/CT scans, while in two patients, PET/CT scans started at the skull base. PET/CT scans in all patients included images from skull base to mid-thigh and all patients received [^18^F]FDG-PET/CT 63.8 ± 9.9 min after injection. Following completion of the FDG-PET scan, patients were allowed to get off the scanner and, after a short break, [^68^Ga]Ga-FAPI-46 was injected. Subsequent dual-tracer PET/CT was performed during the same medical appointment, 13.9 ± 12.3 min after additional injection of [^68^Ga]Ga-FAPI-46. The time interval between injection of [^18^F]FDG and [^68^Ga]FAPI was 104.5 ± 20.2 min, the interval between the two scans was 54.6 ± 27.8 min (Table 1). To ensure an exact correlation between the PET/CT for treatment planning and subsequent radiotherapy, all patients wore either a long thermoplastic mask (patients with head and neck cancers) or immobilization devices for head, arms, back, and knees (patients with esophageal cancer). Two independent reviewers visually identified all pathological findings on single-tracer and dual-tracer PET/CT, recording and comparing the number of lesions and localizations of pathologies. In order to correlate findings and to exclude unspecific findings, a correlation with CT scans was performed.

**Table 1:** Patient characteristics and scan data

Patients (*n* = 19)	Number	
**Age (years); mean, SD**	65.2 ± 10.8	
**Sex**	Male 15; female 4	
**Malignant findings PET/CT**	Single-tracer	Dual-tracer
**Tumors; *n* (*N*** **=** **20)**	18	20
*Oropharyngeal CA*	3^a^	3^a^
*Cancer of mouth floor*	3^a^	3^a^
*Hypopharyngeal CA*	2	2
*Laryngeal CA*	1	1
*Esophageal CA*	9	11
Cervical	13	13^b^
Mediastinal	3	3^b^
Mesenterial	0	1
*Liver metastasis*	1	1
Time between injection FAPI and scan (min); mean, SD	13.9 ± 12.3	
Time between FDG and FAPI Scan (min); mean, SD	54.6 ± 27.8	

^a^Includes one patient with cancer of the oropharynx and cancer of the mouth floor

^b^Additional suspicious lymph node detected

### Target volume delineation

The Eclipse treatment planning system (TPS; Varian Medical Systems; Siemens Healthineers, Erlangen, Germany) was used for target volume delineation and matching of the present imaging. By ensuring that the positioning for scanning of all patients was the same as that used for their subsequent radiotherapy, we were able to achieve exact matching of the images. Radiotherapy reference points were marked on the patients’ skin.

For comparison of target volume delineations, two GTVs were created independently. First, a GTV based exclusively on [^18^F]FDG PET/CT was created (_single-tracerPET_GTV) and, second, a GTV based on the [^18^F]FDG&[^68^Ga]Ga-FAPI-PET/CT was set up (_dual-tracerPET_GTV). For PET/CT based contouring, a window of SUVmax 0–5 was employed. The final GTVs of head and neck cancers were defined in accordance with the latest EORTC contouring guidelines for HNCs, GTVs for esophageal cancer were contoured according to the practical guideline of the expert consensus group for contouring of esophageal cancer [20, 21] and approved by a board-certified radiation oncologist.

Potential pitfalls or known benign sites of FAP uptake were taken into consideration when interpreting discrepant tumor areas (FDG/FAPI+ but FDG–) [22]. Most importantly, lesions were correlated with CT imaging to confirm FDG- or FAPI-positive findings. No upstaging or treatment decisions were based on [^18^F]FDG&[^68^Ga]Ga-FAPI-46-PET/CT information alone. Indeed, all such decisions took the results of clinical examination and CT and MRI imaging into consideration and were discussed in multidisciplinary meetings with clinicians (otolaryngologists, gastroenterologists), physicians for nuclear medicine, and radiation oncologists in consensus.

### Tumor-to-background ratios and functional tumor volume measurements

Volumes of interest (VOIs) were drawn around the primary tumor and, if present, the metastases, to determine the maximum count rates (CRmax) and/or peak count rates (CRpeak) of the lesions. Mean count rates (CRmean) of reference tissue (cerebellum, mediastinal blood pool and liver) were measured with VOIs of 1 cm diameter in the cerebellum and in the descending thoracic aorta (representing mediastinal blood pool), and VOIs of 2 cm diameter in the right liver lobe.

The TBRs were measured by ratios of CR between suspicious lesions and reference tissue obtained from both [^18^F]FDG-PET/CT and [^18^F]FDG&[^68^Ga]Ga-FAPI-46-PET/CT. Ratios of SUVmax/SUVmean and SUVpeak/SUVmean were calculated for single- and dual-tracer PET/CT. In two patients, TBRs with cerebellum as reference tissue could not be measured properly since the cerebellum was not fully included in the scan field.

The FTV was measured in the single- and dual-tracer PET/CT scans using the automatized lesion-detection tool from SyngoVia (Siemens Healthineers, Erlangen, Germany). First, SUVmax for the aorta was obtained from a spherical 1‑cm VOI in the descending thoracic aorta. Secondly, SUVmax was measured within all tumor sites with increased tracer uptake ([^18^F]FDG and [^18^F]FDG&[^68^Ga]Ga-FAPI-46). Manual corrections were performed in cases where nonmalignant tissue was included in the automatic calculation. Measurements were performed as total FTV including primary tumor and metastasis (FTV_total_) as well as FTV_tumour_ and FTV_metastasis_ measurements of primary tumor and metastasis separately. A threshold of 41% of the SUVmax within the respective tumor site (FTV41%) was used for FTV calculations.

All procedures were performed according to the regulations of the local authorities (district administration of Cologne, Germany) and the requirements for performance of this retrospective analysis were verified by the local institutional review board (University of Cologne). This retrospective study was carried out in accordance with the Declaration of Helsinki, with the written consent of all patients to PET/CT imaging and inclusion of their data for scientific analysis.

Descriptive statistics were used to present patient characteristics and results. A Wilcoxon matched-pairs signed-rank test was performed to check for significant differences between continuous variables. A *p*-value of less than 0.05 (*p* < 0.05) was regarded as statistically significant. Pearson’s correlation coefficient was used to measure the strength of the correlation. All statistical analyses were performed using SPSS Statistics v.28 (IBM Corp., Armonk, NY, USA).

## Results

### Patient cohort, PET/CT scan parameters, and lesion detection

In total, PET/CTs of 19 male (*n* = 15) and female (*n* = 4) patients with an average age of 65.2 ± 10.8 years (range 47–86 years) were evaluated for this study. Eight patients were referred from an ear, nose, and throat clinic, with two patients suffering from oropharyngeal carcinoma, two patients with cancer of the mouth floor, two patients with hypopharyngeal, and one patient with laryngeal cancer. One patient with oropharyngeal carcinoma had additional floor of the mouth cancer, while 11 patients suffered from esophageal cancer (Table 1). Lymph node (LN) metastasis was found in eight patients, while one patient had LN metastasis and liver metastasis.

Both single- and dual-tracer PET/CT were tolerated well by all patients, without any record of adverse reactions or side effects. All patients first received [^18^F]-FDG-PET/CT with an average activity of 233.6 ± 43.6 MBq [^18^F]-FDG. At an interval of 40.7 ± 22 min after [^18^F]-FDG-PET/CT, patients were injected intravenously with approximately 164.3 ± 33.5 MBq of [^68^Ga]Ga-FAPI-46. Whole-body PET/CT was then run with both agents 13.9 ± 12.3 min after injection of [^68^Ga]Ga-FAPI-46.

**Fig. 1:**
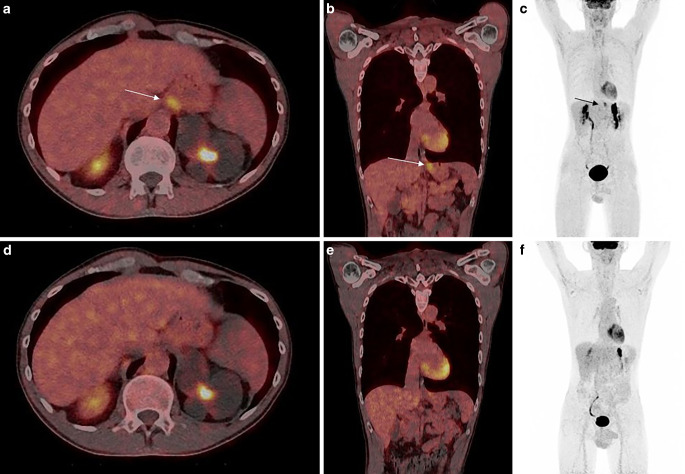
Transverse (**a**) and (**b**) coronal section of a fused dual-tracer [^18^F]FDG&[^68^Ga]Ga-FAPI-46-PET/CT and maximum intensity projection (*MIP*) in (**c**) show mild tracer uptake at the gastroesophageal junction, marked with *white* and *black arrows*. No tracer accumulation could be visualized in the same anatomic region after single-tracer PET/CT using [^18^F]FDG in fused images viewed in the axial (**d**) or coronal plane (**e**) or on MIP (**f**). Physiologic enhancement of the brain, myocardial, and urogenital systems is visible in both scans

All primary tumors could be clearly detected in [^18^F]FDG&[^68^Ga]Ga-FAPI-46-PET/CT, whereas in one patient with cancer of the gastroesophageal junction (Fig. 1) and another patient with esophageal cancer in the mid-third of the esophagus, the primary tumor could not or just roughly be visualized with [^18^F]FDG-PET/CT. In one patient with esophageal cancer, [^18^F]FDG-PET/CT revealed metastasis in only one mediastinal lymph node, whereas [^18^F]FDG&[^68^Ga]Ga-FAPI-46-PET/CT showed tracer accumulation in two additional lymph nodes of the same drainage region. In a different patient with known cervical LN metastasis, an additional suspicious cervical LN was found with dual-tracer PET/CT, which showed no tracer uptake on single-tracer [^18^F]FDG-PET/CT. One mediastinal lymph node of a different patient with esophageal cancer displayed a discrete non-suspicious tracer accumulation on [^18^F]FDG-PET/CT but a suspiciously high accumulation on [^18^F]FDG&[^68^Ga]Ga-FAPI-46-PET/CT; a similar enhancement pattern was seen in a different patient in a cervical LN.

Unspecific tracer accumulation was detected in dual-tracer PET/CT around joints (hip and shoulder), both intramuscularly and in tendons, and was evaluated as degenerative joint changes, bursitis, or tendinopathy. A presumably unspecific subcapsular tracer accumulation in the left liver lobe was detected with dual-tracer PET/CT, most likely as a correlate of a reactive process, with unremarkable follow-up CTs. In one patient, unspecific tracer accumulation was seen in the vein angle. The strong liver enhancement in another patient on [^18^F]FDG&[^68^Ga]Ga-FAPI-46-PET/CT correlated with a hepatomegaly. Imaging and anamnesis of the patient with extensive and regular consumption of alcohol and nicotine led to suspicion of liver cirrhosis.

### GTVs measured on dual-tracer PET/CT are larger in all patients compared to GTVs of single-tracer PET/CT

**Fig. 2:**
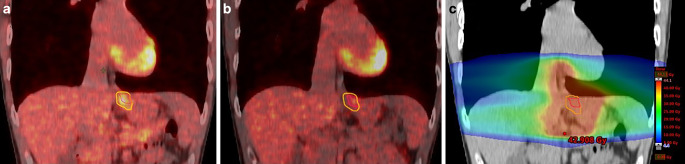
Neoadjuvant chemoradiation (radiotherapy with 41.4 Gy given in 23 fractions of 1.8 Gy and concomitant intravenous carboplatin [AUC 2 mg/mL/min] plus intravenous paclitaxel [50 mg/m^2^ of body surface area]) in a patient with early stage esophageal cancer. **a** Coronal view, dual-tracer PET scan, *yellow line* _dual-tracerPET_GTV, *red line* _single-tracerPET_GTV; **b** Coronal view, same anatomic region and same GTV delineations in the single-tracer PET scan, *yellow line* _dual-tracerPET_GTV, *red line* _single-tracerPET_GTV; **c** Coronal view of radiotherapy plan, colorwash dose distribution

**Fig. 3:**
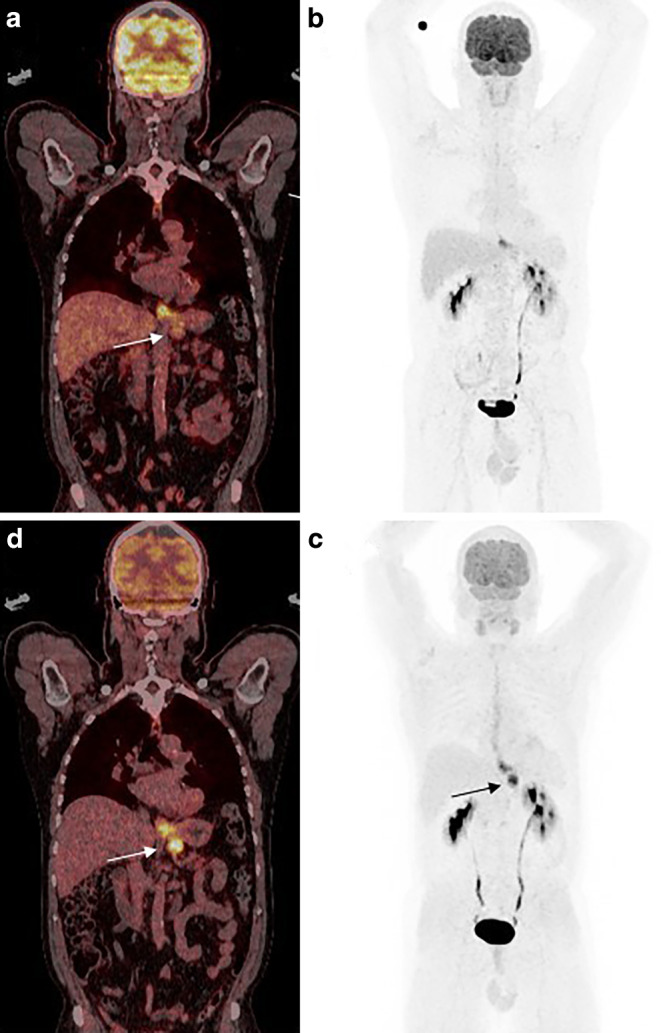
**a** Coronal section of a fused single-tracer [^18^F]FDG-PET/CT and maximum intensity projection (MIP) in (**b**) show a tracer uptake in the gastroesophageal junction with suspicious enlarged mesenterial lymph node without tracer accumulation (*arrow*). Tracer accumulation of the tumor and suspicious lymph node (*arrow*) could be visualized with dual-tracer PET/CT using [^18^F]FDG&[^68^Ga]Ga-FAPI-46 in fused images viewed in the coronal plane (**c**) or on MIP (**d**). Physiologic enhancement and liver are visible in both scans

**Fig. 4:**
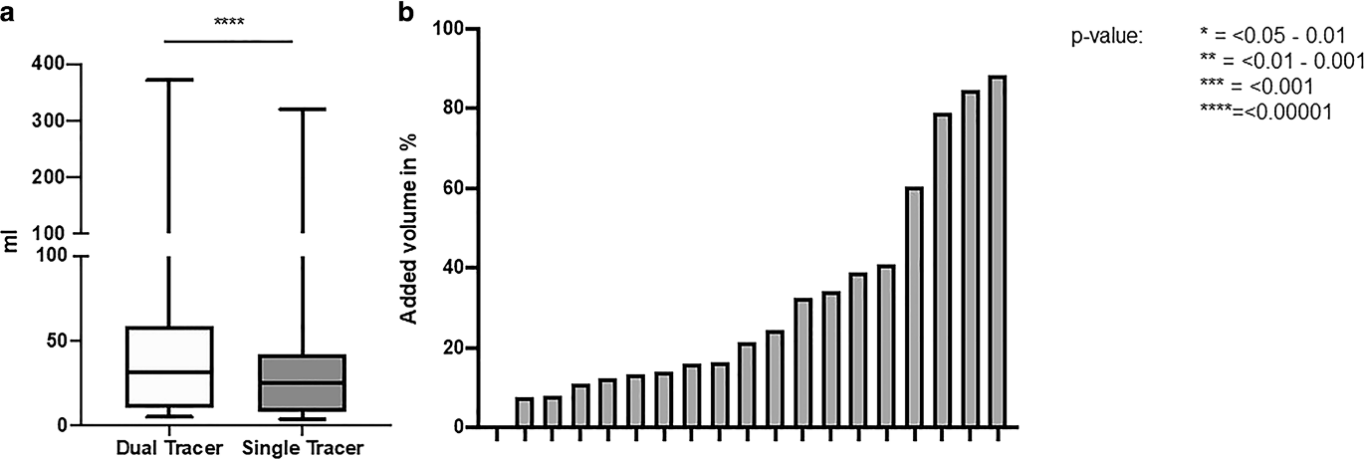
Greater gross tumor volume (GTV) measured with dual-tracer PET/CT as compared to single-tracer PET/CT. **a** Boxplot showing significant differences between _dual-tracerPET_GTV and _single-tracerPET_GTV. **b** Diagram of percentage of added tumor volumes due to larger _dual-tracerPET_GTV compared to _single-tracerPET_GTV

Patients in this cohort were treated with neoadjuvant or definitive chemoradiation for esophageal or head and neck cancer. We performed GTV measurements in all patients with single- and dual-tracer PET/CT. In two patients, contouring GTV on the basis of the single-tracer PET/CT was impaired by low FDG accumulation compared to background tissue. Even when acknowledging endoscopic examinations, contrast-enhanced CT, and MRI, insecurities about the GTV delineation remained and local spread could only be determined in dual-tracer PET/CT. A broad range of GTV sizes was found with _single-tracerPET_GTV (between 3.8 and 321 ml; mean 41.8 ± 71 ml) and, significantly larger, with _dual-tracerPET_GTV (between 5 and 372 ml; mean 51.2 ± 82.2 ml; Table 2). The _dual-tracerPET_GTVs were larger in all patients compared to _single-tracerPET_GTVs (Fig. 2), the additional volume measured with dual-tracer PET/CT being between 0.01 and 51.2 ml (mean 9.3 ± 11.8 ml) and 0.2 and 88.3% (mean 31.8 ± 27.3%; Table 2, Fig. 3). Correct delineation of the GTV had an immediate impact on the clinical target volume (CTV) and boost planning (Fig. 4).

**Table 2:** Absolute values of GTV measurements (*n* = 19): _dual-tracerPET_GTV, _single-tracerPET_GTV, and the difference between the two (_dual-tracerPET_GTV-_single-tracerPET_GTV) in millimeter and percentage

	_Dual-tracerPET_GTV (ml)	_Single-tracerPET_GTV (ml)	Difference (ml)	Difference (%)
Mean (SD)	51.2 (82.2)	41.8 (71.0)	9.3 (11.8)	31.8 (27.3)
Median (Min, Max)	31.2 (5.01, 372)	25.1 (3.8, 321)	5.5 (0.01, 51.2)	21.4 (0.2, 88.3)

Significant differences between _dual-tracerPET_GTV and _single-tracerPET_GTV

### Mean TBRs of tumors and metastases in dual-tracer PET/CTs are mostly higher compared to single-tracer PET/CT using CRmax

**Fig. 5:**
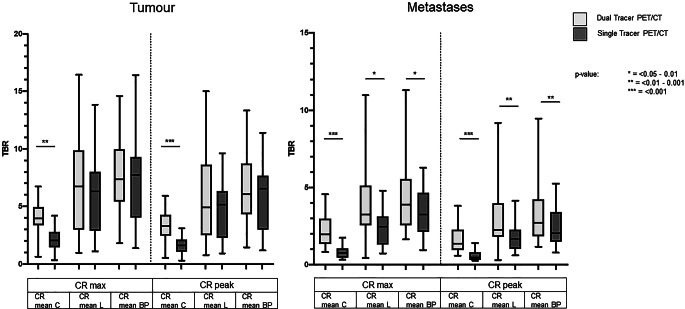
Boxplot of tumor-to-background ratios (*TBRs*) measured on single- and dual-tracer PET/CT showing significantly larger mean TBRs of tumors compared to cerebellum (*C*) on dual-tracer PET/CT and slightly higher mean TBRs compared to liver tissue (*L*) using CRmax measurements but not with CRpeak. Mean TBRs of tumors compared to blood pool (*BP*) were slightly higher with single- compared to dual-tracer PET/CT. TBRs of metastases were significantly higher with dual-tracer vs. single-tracer PET/CT compared to all reference regions using CRmax as well as CRpeak

As shown in Fig. 5 and Table 3, TBRs of tumors and metastases with reference tissue cerebellum were significantly higher in [^18^F]FDG&[^68^Ga]Ga-FAPI-46-PET/CT compared to [^18^F]FDG-PET/CT. Mean TBRs measured between tumors and liver were slightly higher with dual-tracer PET/CT using CRmax and slightly lower compared to single-tracer PET/CT using CRpeak measurements. Mean TBRs between tumors and mediastinal blood pool were lower in dual- compared to single-tracer PET/CT, but the difference did not reach significance. Significant differences with higher mean TBR of metastasis compared to mediastinal blood pool could be shown using CRmax and CRpeak as measurement parameters, whereas significant differences between mean TBR of metastasis and liver tissue were only found using CRmax measurements (Table 3, Fig. 5).

**Table 3:** Target-to-background ratios (*TBRs*) of count rates (*CRs*) measured in tumors, metastases, and background on [^18^F]-FDG&[^68^Ga]Ga-FAPI-46-PET/CT and [^18^F]-FDG-PET/CT

TBR	[^18^F]-FDG&[^68^Ga]Ga-FAPI-46-PET/CT (mean ± SD)	[^18^F]-FDG-PET/CT (mean ± SD)	Wilcoxon signed-rank test; Pearson’s correlation
*CR*_*Tumor*_*/CR*_*Background*_			
CRmax_T_/CRmean_C_	3.9 ± 1.6	2.1 ± 1	*p* = 0.003 r = 0.589
CRmax_T_/CRmean_L_	7.3 ± 4.2	6.2 ± 3.2	*p* = 0.171 r = 0.520
CRmax_T_/CRmean_BP_	7.8 ± 3.5	8.1 ± 4.2	*p* = 0.573 r = 0.511
CRpeak_T_/CRmean_C_	3.2 ± 1.4	1.7 ± 0.8	*p* < 0.001 r = 0.744
CRpeak_T_/CRmean_L_	6.0 ± 3.6	4.8 ± 2.3	*p* = 0.117 r = 0.622
CRpeak_T_/CRmean_BP_	6.5 ± 3	6.2 ± 2.9	*p* = 0.376 r = 0.644
*CR*_*Metastasis*_*/CR*_*Background*_			
CRmax_M_/CRmean_C_	2.2 ± 1	0.8 ± 0.4	*p* < 0.001 r = 0.815
CRmax_M_/CRmean_L_	3.6 ± 2.3	2.5 ± 1.2	*p* = 0.010 r = 0.596
CRmax_M_/CRmean_BP_	4.4 ± 2.4	3.4 ± 1.6	*p* = 0.020 r = 0.762
CRpeak_M_/CRmean_C_	1.7 ± 0.9	0.6 ± 0.4	*p* < 0.001 r = 0.882
CRpeak_M_/CRmean_L_	2.9 ± 2	1.9 ± 1.1	*p* = 0.006 r = 0.671
CRpeak_M_/CRmean_BP_	3.4 ± 2	2.5 ± 1.4	*p* = 0.008 r = 0.851

### FTV of total tumor volume and primary tumors are significantly larger in [^18^F]FDG&[^68^Ga]Ga-FAPI-46-PET/CT compared to [^18^F]FDG-PET/CT

**Fig. 6:**
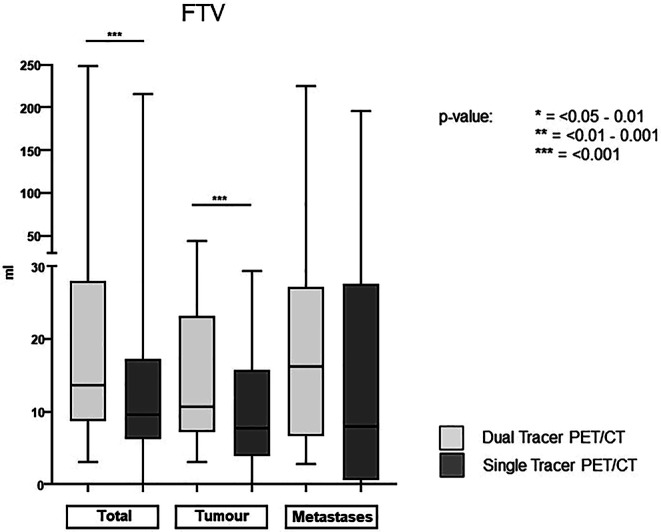
Boxplot of functional tumor volumes (*FTV*) measured on single- and dual-tracer PET/CT showing significantly higher volumes with [^18^F]FDG&[^68^Ga]Ga-FAPI-46-PET/CT compared to PET/CT after injection of [^18^F]FDG only. Although mean FTVs of metastases were generally larger on dual- compared to single-tracer PET/CT, the differences were not significant

Automatized FTV measurement was possible in all dual-tracer scans. In contrast, two esophageal cancers could not be detected with SyngoVia lesion scout, either because there was no FDG accumulation or because it was so mild as to fall below that of the background tissue (Fig. 1). FTV measurements of primary tumors only (FTV_tumour_) and total tumor volume (tumor and metastasis, FTV_total_) were significantly larger in dual-tracer PET/CT compared to single-tracer PET/CT. FTV_total_ was 58.5 ± 80.6 ml in dual-tracer PET/CT compared to 43.3 ± 72.8 ml on the single-tracer scan. Mean FTV_tumor_ measured 19.7 ± 13.2 in dual-tracer and 12.8 ± 10.6 ml in single-tracer PET/CT. Although not significant, mean FTV_metastasis_ was much larger with 41.2 ± 75 ml in [^18^F]FDG&[^68^Ga]Ga-FAPI-46-PET/CT compared to 32.9 ± 66.6 ml in [^18^F]FDG-PET/CT (Table 4, Fig. 6).

**Table 4:** Functional tissue volume (*FTV*) and total malignant tissue (*FTV*_*total*_), tumour (*FTV*_*tumor*_), metastasis (*FTV*_*metastases*_) on [^18^F]-FDG&[^68^Ga]Ga-FAPI-46-PET/CT and [^18^F]-FDG-PET/CT

FTV	[^18^F]-FDG&[^68^Ga]Ga-FAPI-46-PET/CT (mean ± SD)	[^18^F]-FDG-PET/CT (mean ± SD)	Wilcoxon signed-rank test; Pearson’s correlation
FTV_total_	58.5 ± 80.6	43.3 ± 72.8	*p* < 0.001 r = 0.986
FTV_tumour_	19.7 ± 13.2	12.8 ± 10.6	*p* < 0.001 r = 0.922
FTV_metastases_	41.2 ± 75	32.6 ± 66.6	*p* = 0.093 r = 0.993

## Discussion

The present study demonstrates that malignant lesions (primary tumors and metastases) can be evaluated equally well and in some cases even better using dual-tracer PET/CT with [^18^F]FDG and [^68^Ga]Ga-FAPI-46 compared to single-tracer PET/CT with [^18^F]FDG. An equal performance in 13 patients and a better diagnostic performance of dual-tracer PET/CT with [^18^F]FDG&[^68^Ga]Ga-FAPI-46 compared to [^18^F]FDG was shown in six patients. In two patients, the tumor was only visible with dual-tracer PET/CT, while in another four patients, additional lesions suspicious for lymph node metastases were found with [^18^F]FDG&[^68^Ga]Ga-FAPI-46-PET/CT compared to [^18^F]FDG-PET/CT. A superior diagnostic performance of [^68^Ga]Ga-FAPI-PET/CT or [^68^Ga]Ga-FAPI-PET/MRI compared to [^18^F]FDG-PET/CT has recently been described for several tumor entities [9, 23, 24], mostly caused by higher tumor-to-background ratios, especially (but not only) for lesions located in tissues with higher background tracer uptake levels of FDG such as liver and brain tissue. Similar results could be reproduced in a preliminary study comparing [^18^F]FDG&[^68^Ga]Ga-FAPI-46 dual-tracer PET/CT and single-tracer PET/CT with [^18^F]FDG in patients with HNT and esophageal cancer [19]. The present study underlines the findings of the preliminary study, showing equal or even better diagnostic performance of dual-tracer PET/CT compared to single-tracer PET/CT. An explanation for the improved visualization of malignant tissue with [^68^Ga]Ga-FAPI may be found in the heterogeneous composition of tumor lesions. The higher amounts of tumor stroma compared to tumor cells could explain the supplementary effect of [^68^Ga]Ga-FAPI when added to [^18^F]FDG, since the stroma contain FAP-expressing CAFs.

The advantage of dual-tracer PET/CT compared to single-tracer PET/CT arises from the fact that different portions of the tumor, i.e., the CAFs in the peritumoural stroma, are displayed with [^68^Ga]Ga-FAPI, which might be only slightly FDG positive or FDG negative, thus leading to better visualization of some tumor entities and metastases [25, 26]. On the other hand, FAPI-negative but FDG-positive lesions as described in a lymphoma patient or a melanoma patient [27, 28] would not be missed using dual-tracer PET/CT, since accumulation of both tracers is displayed with this protocol.

In addition, a potential summative effect of the two tracers in tissue portions showing uptake of both tracers might explain why small malignant structures such as small tumors and lymph nodes are better visualized with dual-tracer compared to single-tracer PET/CT in this study. We assume that this summative effect might be of help to display smaller metastases. In contrast to our previous study comparing dual- and single-tracer PET/CT, the differences between the two scans in terms of TBRs measured with reference tissue liver and blood pool were less distinct in the current study. A possible reason for this might be the shorter time period between the two scans and a shorter time period between [^68^Ga]Ga-FAPI-46 injection and image acquisition in the present study.

The high potential of FAPI for staging and tumor characterization has recently been described in sarcoma patients. In addition, a close correlation between the intensity of FAPI tumor uptake and PET and histopathological FAP expression was shown in sarcoma patients [29], underlining the notion that FAP expression of tumors can be reliably displayed in vivo with FAPI-PET. This is of importance, since a meta-analysis evaluating histologic FAP expression in various tumors showed significant correlation between high FAP expression of tumors and tumor progression as well as overall survival [15]. FAPI-PET would therefore not only be an alternative to FDG-PET for tumor diagnostics and staging, but may also hold great potential as a tool for treatment decisions and prognostic evaluation.

FDG-PET/CT allows assessment of the metabolic tumor volume (MTV), which has been shown to be a prognostic marker for outcome in patients with HNT [30] and esophageal cancer [31, 32]. As with MTV, we measured the FTV of malignant tissue in single- and dual-tracer PET/CT and could show a significantly larger FTV of primary tumor tissue and of total malignant tissue as well as a trend towards larger FTV in metastatic tissue. A reason for the less distinct difference in metastatic tissue may lie in the (still) lower volume of CAF-expressing tumor stroma in metastases as compared to primary tumor. Whether the FTV of dual-tracer PET can be used as a prognostic marker for prediction of outcome will need to be investigated in further studies.

As described above, GTV measurements could be performed without any problem on dual-tracer PET/CT and led to calculation of a significantly larger tumor volume compared to single-tracer PET/CT. This is in line with reports from the literature, which describe larger GTVs and higher TBRs in PET/CT with Ga-FAPI compared to FDG [12] as well as higher tracer uptake and better detection of metastatic lymph nodes with FAPI compared to FDG [33]. So far, the roles of CAFs in the tumor microenvironment are not entirely understood, since they can promote antitumorigenic effects as well as initiating tumor progression and invasiveness [34]. Studies of the effects of radiation on CAFs show a controversial impact of radiation on the tumor-promoting abilities of CAFs, with enhanced and diminished protumorigenic potential of treated CAFs [35]. Although imaging of CAFs with [^68^Ga]Ga-FAPI-PET/CT shows promising results for cancer staging, it remains to be clarified whether radiation treatment planning based on [^68^Ga]Ga-FAPI-PET/CT can provide additional value for treatment planning beyond improved localization of suspicious lesions and assessment of tumor extension.

The results of this study indicate that dual-tracer PET/CT with [^18^F]FDG and [^68^Ga]Ga-FAPI-46 is comparable and in some cases potentially superior for detection of malignant tissue (as compared to single-tracer FDG-PET) and that this dual-tracer scan results in calculation of larger FTVs and GTVs. This latter effect is most probably due to the complementary strengths of both tracers, displaying different cells and/or tissue compartments of the tumor and metastasis. The single-session dual-tracer protocol opens up opportunities by enabling cancer patients to receive a multimodal diagnostic workup with consecutively higher sensitivity within the same medical appointment. Limitations of this approach could be the failure to define exclusively FAPI-positive lesions and a mixed measurement of FDG and FAPI in malignant tissue FTV. A second limitation could be regarding false-positive findings of FAPI-PET/CT, although it is highly unlikely that these are misinterpreted by the interdisciplinary panel consisting of nuclear medicine physicians, radiologists, and radiation oncology physicians, but cannot be excluded. However, if the main focus is on achieving optimal diagnostic sensitivity, these limitations may be of minor importance. In that case, a simplified protocol could be implemented based on dual-tracer injection but only a single PET/CT acquisition (i.e., skipping the FDG-only scan and therefore further shortening the protocol). Such concepts should be evaluated in future studies.

## Conclusion

The present study demonstrates that dual-tracer PET/CT with [^18^F]FDG&[^68^Ga]Ga-FAPI-46 shows an equal and, in a third of patients, even better diagnostic performance in cancer staging compared to exclusive [^18^F]FDG-PET/CT imaging. In addition, significantly larger functional and gross tumor volumes (FTVs and GTVs) were calculated on the basis of the dual-tracer compared to single-tracer PET/CT findings. This might in part be due to higher tumor-to-background ratios and expression of FAP in different portions of the tumor compared to FDG as well as to summative effects of the two tracers.

While our knowledge of imaging with [^68^Ga]Ga-FAPI-46-PET/CT is still growing, dual PET/CT with [^18^F]FDG&[^68^Ga]Ga-FAPI-46 allows combination of the established [^18^F]FDG-PET/CT standard procedure with [^18^F]FDG&[^68^Ga]Ga-FAPI-46-PET/CT within one appointment. This provides additional diagnostic benefit without significantly increasing the burden of multiple diagnostic procedures on cancer patients.
